# Many Ribosomal Protein Genes Are Cancer Genes in Zebrafish

**DOI:** 10.1371/journal.pbio.0020139

**Published:** 2004-05-11

**Authors:** Adam Amsterdam, Kirsten C Sadler, Kevin Lai, Sarah Farrington, Roderick T Bronson, Jacqueline A Lees, Nancy Hopkins

**Affiliations:** **1**Center for Cancer Research, Massachusetts Institute of TechnologyCambridge, MassachusettsUnited States of America; **2**Department of Pathology, Tufts University School of Veterinary MedicineBoston, MassachusettsUnited States of America

## Abstract

We have generated several hundred lines of zebrafish *(Danio rerio),* each heterozygous for a recessive embryonic lethal mutation. Since many tumor suppressor genes are recessive lethals, we screened our colony for lines that display early mortality and/or gross evidence of tumors. We identified 12 lines with elevated cancer incidence. Fish from these lines develop malignant peripheral nerve sheath tumors, and in some cases also other tumor types, with moderate to very high frequencies. Surprisingly, 11 of the 12 lines were each heterozygous for a mutation in a different ribosomal protein (RP) gene, while one line was heterozygous for a mutation in a zebrafish paralog of the human and mouse tumor suppressor gene, neurofibromatosis type 2. Our findings suggest that many RP genes may act as haploinsufficient tumor suppressors in fish. Many RP genes might also be cancer genes in humans, where their role in tumorigenesis could easily have escaped detection up to now.

## Abstract 2

A screen for tumour suppressor genes in zebrafish has identified several genes encoding ribosomal proteins, indicating that these genes might be an unappreciated class of cancer genes

## Introduction

The zebrafish *(Danio rerio)* has long been used as a model organism for the identification of genes required for early vertebrate development (Kimmel 1989). There is reason to believe that the zebrafish can also be used in genetic screens to identify cancer genes. Zebrafish can live for 4–5 y (Gerhard et al. 2002), and like other fish species (Schmale et al. 1986; Wittbrodt et al. 1989), they develop tumors in a variety of tissues (Amatruda and Zon 2002; Smolowitz et al. 2002). They are also susceptible to chemical carcinogens and to well-known oncogenes, in a manner similar to the conventional mouse models (Beckwith et al. 2000; Spitsbergen et al. 2000a, 2000b; Langenau et al. 2003). Many of the spontaneous and chemically- or oncogene-induced tumor types are histologically similar to their mammalian counterparts (Amatruda and Zon 2002; Langenau et al. 2003).

The normal functions of many mammalian tumor suppressor genes are required for normal development (Jacks 1996). In fact, nonessential tumor suppressors, such as *p53* (Donehower et al. 1992), appear to be the exception rather than the rule. These findings raised the possibility that one could discover genes with a role in tumorigenesis among zebrafish genes identified initially for having essential roles during embryonic development. We have used retroviral vectors as a mutagen in a large-scale insertional mutagenesis screen and have isolated many zebrafish mutants with lesions in genes essential for embryogenesis (Amsterdam et al. 1999; Golling et al. 2002). We are maintaining approximately 500 lines, in most of which an embryonic lethal mutation is linked to a single proviral insert. We have identified the mutated genes in over 400 of the lines, and these include mutations in 300 distinct zebrafish genes. To maintain the lines, we identify approximately 15 heterozygous carriers and outcross these at 15–20 mo of age to produce the subsequent generation. The maintenance of these mutations in adults provides a unique opportunity to ask whether heterozygosity in genes required for embryonic development predisposes the animals to cancer. Here we describe how such an analysis has identified genes that encode ribosomal proteins (RPs) as cancer genes in zebrafish.

## Results

### Mutations in Many RP Genes Predispose Zebrafish to Malignant Peripheral Nerve Sheath Tumors and Other Cancers

**Figure 1:**
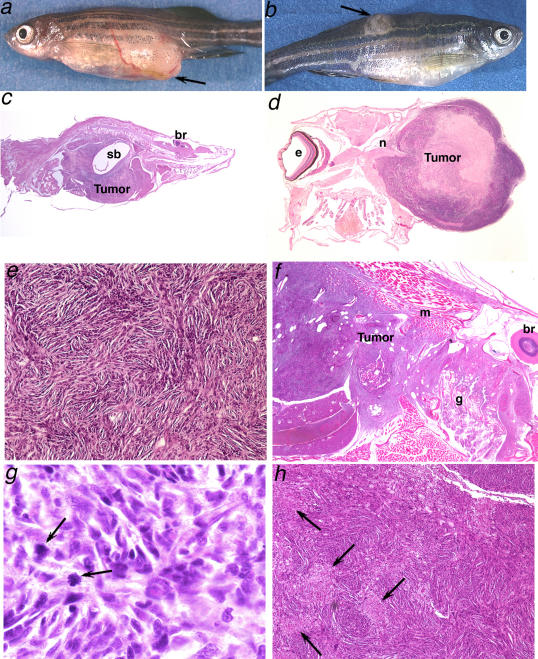
Spindle Cell Tumors Resembling MPNSTs in Zebrafish Heterozygous for Mutations in RP Genes (A and B) Fish with apparent masses, as indicated by the arrows, or other evident pathology were selected for histological analysis: (A) a hi2582 fish, (B) a hi1034B fish. (C–H) Histopathology of representative tumors stained with hematoxylin and eosin reveals patterns consistent with the diagnosis of MPNST in hi10 fish (C and D), hi1974 fish (E–G), and hi1807 fish (H). (C) Tumors typically filled the entire abdomen (sb, swim bladder; br, brain) (80×). (D) A large tumor with central necrosis is seen emanating from the optic nerve (n) (e, eye) (20×). (E) Tumors consist of spindle cells that stack into short fascicles, typically organizing into whorls (400×). (F) Tumor is aggressively invading muscle (m) and gill (g) (br, brain) (100×). (G) Mitotic figures (arrows) are evident (1000×). (H) Areas of focal necrosis (arrows) are frequently seen (200×).

In the course of establishing and maintaining heterozygous mutant lines of fish, we noticed several lines that displayed early mortality by 2 y of age, and this phenotype was seen in successive generations. Typically, only about 10% to 15% of the fish in a tank are lost by 2 y of age, but in these apparently high-mortality lines losses sometimes exceeded 50%. Furthermore, fish from these lines were often found to have gross lumps (Figure 1A and 1B). Histological analysis of step sections showed that the growths were predominantly large, malignant spindle cell tumors that were highly invasive, had a high mitotic index, and often exhibited focal necrosis (Figure 1C–1H). The tumor cells were aligned into stacks and fascicles to form a whirling, storiform pattern (Figure 1E–1H) that resembled malignant peripheral nerve sheath tumors (MPNSTs) seen in other species of fish (Schmale et al. 1983; Roberts 2001) and in mammals (Cichowski et al. 1999; Jimenez-Heffernan et al. 1999; Woodruff 1999). In keeping with the published work on fish tumors, while adhering to the caution suggested by the National Neurofibromatosis Foundation regarding animal models of MPNST (M. McLaughlin, personal communication), we have designated these tumors zMPNSTs (zebrafish MPNSTs).

Although we had occasionally observed individual fish with lumps in our colony, it was unusual to find so many within a single line. Thus, we reasoned that the lines with early mortality that also frequently displayed gross lumps by 2 y of age might be lines with elevated rates of lethal cancer. Surprisingly, we found that the several potentially high-tumor lines were all heterozygous for mutations in genes that encode different RPs. This unexpected observation, combined with the knowledge that many tumor suppressors are recessive embryonic lethal genes, prompted us to survey our colony systematically to determine the incidence and spectrum of tumors arising in the colony as a whole, and to ask specifically whether genes that encode many different RPs predispose to cancer.

To determine cancer incidence in the colony as a whole, we sectioned 152 “control” fish that were 20–26 mo of age. Forty-nine of the control fish were nontransgenic, while 103 were selected at random from 54 lines heterozygous for mutations in genes other than RP genes. The latter fish had been generated and maintained in a comparable manner to our RP mutant lines and thus were appropriate controls. The incidence of tumors detected by step sectioning in this control population was 11% (Table 1D). Although we observed a variety of different tumor types (most frequently seminomas and pancreatic islet cell adenomas), most of the tumors (15/17) were benign neoplasias and none were zMPNSTs. There was neither a significant difference in spectrum nor an increase in incidence of tumors in the non-RP heterozygous mutant fish relative to the wild-type fish, indicating that the presence of viral insertions, per se, does not have an obvious effect on tumorigenicity.

**Table 1:**
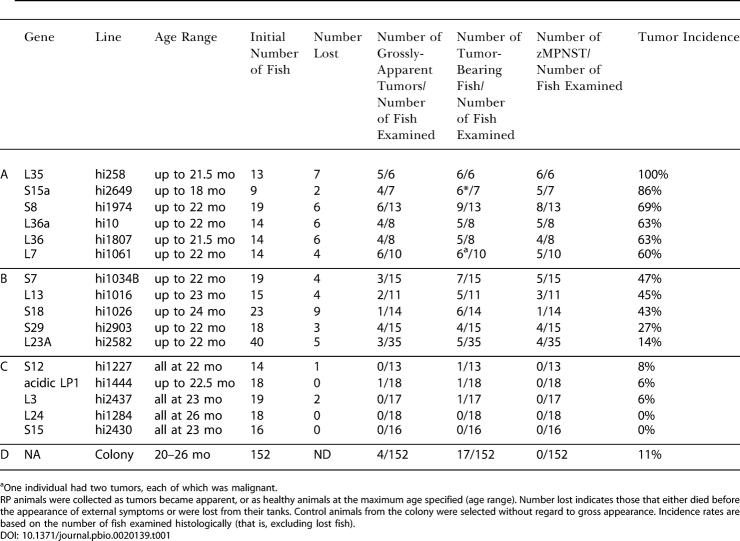
Tumor Incidence in Zebrafish RP-Heterozygous Lines and in the Colony

^a^One individual had two tumors, each of which was malignant. RP animals were collected as tumors became apparent, or as healthy animals at the maximum age specified (age range). Number lost indicates those that either died before the appearance of external symptoms or were lost from their tanks. Control animals from the colony were selected without regard to gross appearance. Incidence rates are based on the number of fish examined histologically (that is, excluding lost fish)

To compare the frequency and types of tumors arising in RP mutant lines with those of the control population, we established the fate of all heterozygotes in a single generation of each of 16 RP mutant lines. In each family, some fish were lost prior to any observation of external symptoms, precluding determination of the cause of death. The rest were sacrificed either when they developed visible masses or when they reached 18 or 22 mo of age, and step sections were examined (Table 1). The 16 RP families fell into three groups with respect to tumor incidence. Six lines had high mortality (including both lost fish and those with external growths) and a high tumor incidence (60% or more, including both fish with gross tumors and tumors detected only upon sectioning). Nearly all of the tumors observed by 22 mo in these lines were zMPNSTs (Table 1A). These lines included those with mutations in RP genes *S8, S15a, L7, L35, L36,* and *L36a.* Five RP mutant lines made up a second group. These lines had either a moderate incidence of cancer, or had a low incidence but were unusual in having an apparently elevated incidence of zMPNSTs. This group included lines with mutations in *L13, L23a, S7, S18,* and *S29.* As in the high-cancer lines, in most lines with moderate cancer incidence, most tumors observed in fish by 22–24 mo of age were zMPNSTs (Table 1B). In one line (hi1026, with a mutation in *S18*), however, other tumor types predominated, suggesting that RP mutations can increase the frequency of tumor types besides zMPNSTs. The third group of RP mutant lines included five lines that were not tumor prone. These lines, with mutations in *L3, L24, LP1, S12,* and *S15,* were indistinguishable from controls in tumor incidence and spectrum (Table 1C versus 1D). In summary, 11 of 16 RP mutant lines had an elevated incidence of cancer, and most of these 11 lines are predisposed to develop zMPNSTs.

**Figure 2:**
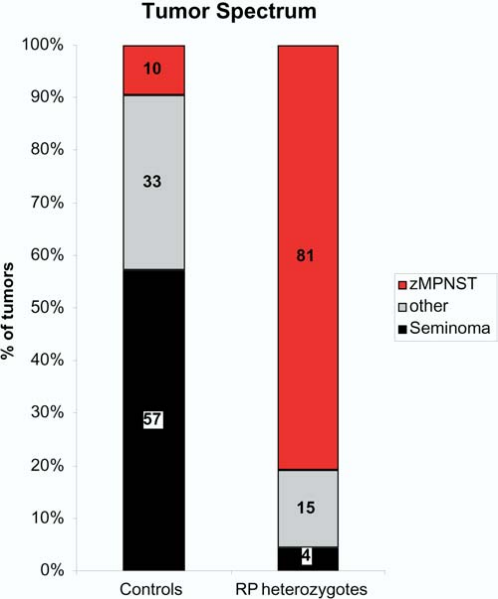
The Tumor Spectrum in Fish Heterozygous for Mutations in RP Genes Shows an Increased Proportion of zMPNSTs Fish with apparent masses were selected and processed for histological analysis. Numbers are shown as percent of the total number of diagnosed tumors from either population. The control group includes 42 tumors from 41 fish, including both wild-type and non-RP family transgenics. The RP group includes 68 tumors from 65 RP heterozygotes from 18 different lines representing mutations in 16 different genes. The “other” tumor category includes pancreatic islet adenomas, ultimobranchial gland tumors, neuroblastomas, retinoblastomas, lymphomas, ganglioneuromas, ductal carcinomas, gastrointestinal adenocarcinomas, hepatocellular carcinomas, leukemias, meningiomas, and histiocytic sarcomas.

Together these findings suggest that zMPNSTs are rare in our colony except in RP mutant lines. However, because the cancer incidence was low in the control fish, we observed only 17 tumors in this group of fish in the experiment described above. Furthermore, only four of these 17 tumors were grossly visible, with 13 being detected only after sectioning. To obtain more data on tumor spectrum in our colony, including the types of tumors that present as externally visible growths in non-RP mutant lines and wild type, we sought out fish with externally visible tumors from throughout our colony, coded them to avoid bias, and identified the tumor types by histological analysis of step sections. In total, we analyzed gross tumors from 41 control fish (wild type or non-RP mutant lines, including the four tumors found above). We also analyzed a total of 65 RP heterozygotes with grossly visible tumors (including the fish represented in Table 1A–1C). Figure 2 shows a comparison of the types of tumors in control versus RP mutant lines that presented as externally visible growths. In the control fish, seminomas accounted for 57% of these tumors, while a wide variety of other tumor types, including ultimobranchial gland tumors, neuroblastomas, islet cell adenomas, and lymphomas, each arose at low frequency. Overall, 69% of the grossly visible tumors observed in non-RP fish were benign. Only 10% of these externally visible tumors were zMPNSTs (see below). In contrast to the control fish, and as is apparent from the data in Table 1, the majority of grossly visible tumors in the RP mutants were zMPNSTs (81%), greatly exceeding the number of seminomas (4%) or other (15%) tumor types (Figure 2). Since fish with external growths were found far more often within RP families than in the colony at large, the dramatic shift in the spectrum of tumors in RP relative to non-RP mutant lines reflects the profound increase in incidence of zMPNSTs rather than any obvious reduction in the incidence of seminomas and other tumor types.

As noted above, we detected zMPNSTs in only four of 41 control fish with grossly visible tumors. Two of these fish, aged 15 and 24.5 mo, were from the hi3332 line, the only non-RP line in which more than a single zMPNST has been observed to date. Significantly, the viral insertion that is linked to the embryonic lethal phenotype of this line lies within one *(NF2a)* of two distinct zebrafish genes that are highly homologous to the mammalian neurofibromatosis type 2 gene *(NF2).* The insertion abrogates expression of this gene in homozygous mutant embryos (Figure S1 and data not shown). *NF2* was originally identified as a tumor suppressor gene that predisposes individuals to develop tumors of the nervous system (Trofatter et al. 1993; Ruttledge et al. 1994). Given this finding, we screened the remaining 53 fish in this family for tumors between 17.5 and 23 mo of age by sectioning. Seven of these 53 fish had small spindle cell tumors. These tumors were not identical to typical zMPNSTs found in RP families, but shared some key characteristics (data not shown). Given the elevated incidence of rare tumor types including zMPNSTs, we conclude that *NF2a* acts as a tumor suppressor gene in fish, as it does in mammals.

### Early Mortality in an RP Mutant Line Results from Multiple Types of Cancer

**Figure 3:**
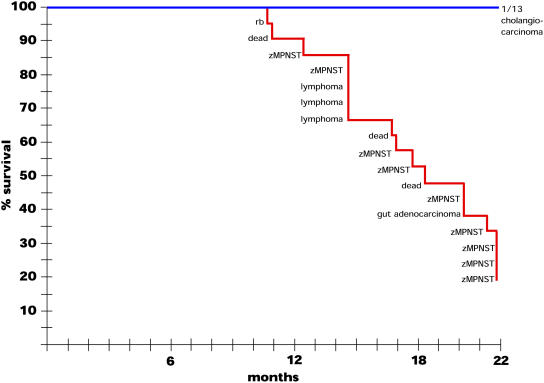
Rate of Tumor Appearance in hi10 Heterozygotes A cohort of 28 hi10 fish and 13 of their noncarrier siblings were observed over 22 mo for the appearance of ill health or externally visible tumors. Symptomatic individuals were sacrificed, fixed, and sectioned for histological analysis. The graph represents the percentage of fish remaining over time, with the diagnosis of each removed fish. Three fish labeled “dead” died before fixation and had too much tissue damage to establish a diagnosis. Also, seven of the carrier fish (though none of the noncarriers) were lost to unknown causes over the course of the experiment; while they most likely died, to be conservative these were removed from the total number of fish charted. At 22 mo, the remaining externally healthy fish (4/21 carriers, 13/13 noncarriers) were also histologically examined, and the status of these fish is indicated.

The experiment described above identified six RP mutant lines with high mortality. While some of the mortality could be accounted for by fish that displayed gross tumors and therefore were removed from the tanks before they died, many fish simply disappeared or were found dead and were too deteriorated to be analyzed histologically. To determine whether early mortality in these lines was entirely due to lethal cancers, and if so, whether it was due to zMPNSTs or to other tumor types, we performed two experiments using fish from the early-mortality, high-tumor hi10 line. In one experiment we screened hi10 heterozygotes and their wild-type siblings weekly for evidence of ill health or externally visible growths in an effort to catch all sick fish before they died or were lost. Sickly fish were sacrificed and subjected to histological examination, as were all of the fish that still appeared healthy at 22 mo of age. The results are shown in Figure 3. Only the RP heterozygous carrier fish displayed early mortality, and, as anticipated, this was due to cancers. Strikingly, among tumors found by 15 mo of age, while two were zMPNSTs, one was a retinoblastoma and three were lymphomas, tumor types that, like zMPNSTs, arise infrequently in our control populations. The tumors detected in the older fish were predominantly zMPNSTs. By the endpoint of the experiment (22 mo) all of the noncarrier sibling controls appeared healthy, and step sectioning detected only one tumor-bearing fish among 13, a frequency comparable to the control population. These results support the conclusion that the early mortality observed in the hi10 line is the result of lethal tumors, and reveal that these include zMPNSTs but also other tumor types.

Further evidence that fish from the hi10 line are predisposed to multiple tumor types was obtained in the second experiment, in which we sectioned hi10 heterozygotes and their noncarrier sibling controls (specifically including any sick or growth-bearing fish along with apparently healthy fish) at approximately six-week intervals between eight and 14 mo of age. As shown in Table 2, we found both grossly visible and occult zMPNSTs and other tumor types in the hi10 carrier fish. Thus, the hi10 line (and presumably other high-mortality RP lines) is predisposed to multiple tumor types, though particularly strongly predisposed to develop zMPNSTs, especially at later time points.

**Table 2:**
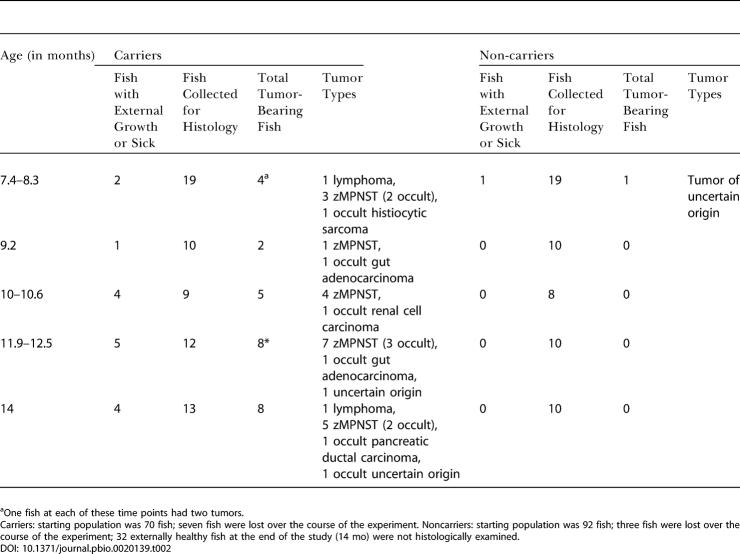
Onset of Tumor Development in hi10 Fish and Noncarrier Siblings

^a^One fish at each of these time points had two tumors

Carriers: starting population was 70 fish; seven fish were lost over the course of the experiment. Noncarriers: starting population was 92 fish; three fish were lost over the course of the experiment; 32 externally healthy fish at the end of the study (14 mo) were not histologically examined

### RP Genes May Be Haploinsufficient Tumor Suppressors

**Figure 4:**
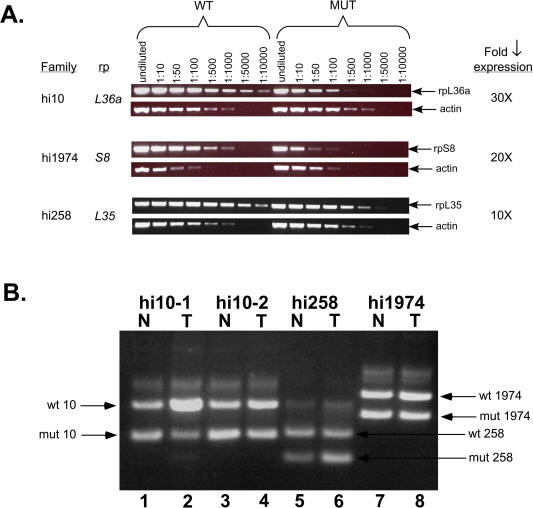
RP Genes Appear to Be Haploinsufficient Tumor Suppressors (A) RP mutations decrease the amount of RP gene expression. RNA was prepared from 3-d-old homozygous mutant embryos and their wild-type siblings, and serial dilutions of first strand cDNA were used as templates for PCR. The decrease in expression in the mutants can be determined by the difference in the dilution between wild type and mutant where the PCR product amount diminishes. The actin control shows that the total amount of mRNA was the same between samples. (B) LOH is not observed in RP mutant tumors. DNA was prepared from tumors (T) and normal tissue (N) from the same fish, and PCR was conducted with three primers that show the presence or absence of both the insert-bearing (mutant) and wild-type chromosomes. In each case, the upper band is the wild-type chromosome and the lower band is the insert-bearing one. hi10 fish #1 normal (lane 1), tumor (lane 2); hi10 fish #2 normal (lane 3), tumor (lane 4); hi258 fish normal (lane 5), tumor (lane 6); hi1974 fish normal (lane 7), tumor (lane 8).

Dominant mutations that predispose vertebrates to cancer can be activated oncogenes, recessive tumor suppressors, or haploinsufficient tumor suppressors (Largaespada 2001). Several lines of evidence suggest that RP mutant genes may be acting as haploinsufficient tumor suppressors in zebrafish. The mutagenic inserts in all of our RP mutant lines reduced or eliminated expression of the RP gene, as determined by RT-PCR and, in some cases, Northern blotting (Figure 4A and data not shown). Thus, most if not all of these viral insertions appear to be loss-of-function mutations. This suggests that the RP genes are not mutated to form activated oncogenes, but rather may act as tumor suppressors. In mammals, the most frequent mechanism of inactivation of recessive tumor suppressor genes is the acquisition of a mutation (either germline or somatic) in one allele and subsequent loss of the wild-type allele through loss of heterozygosity (LOH) (Haber and Harlow 1997). Thus, we investigated whether the wild-type RP gene had been lost in the zebrafish tumors. We isolated both normal and tumor tissue from three RP heterozygous mutant lines, hi10, hi258, and hi1974, each of which showed a reduction in expression of its respective RP mutant gene of 10-fold or more (Figure 4A) and examined DNA from these samples for the presence of the mutant and wild-type RP alleles by PCR (Figure 4B). In every case, we detected the wild-type allele, arguing against LOH in these tumors. A concern is that tissue contamination can yield misleading LOH results, particularly because the red blood cells of fish are nucleated. Thus control PCR experiments were performed in which DNA samples from heterozygous and homozygous embryos were mixed at different ratios. The results show that our assay was sensitive to a decrease as small as 3-fold in the relative amount of the wild-type allele (data not shown). Thus, unless the tumor samples contained more than 33% nontumor cells, we can conclude that the wild-type RP alleles were not lost in these tumors and thus the RP genes are probably not recessive tumor suppressors. In one of the tumor samples shown in Figure 4, tumor hi10–1, the wild-type allele appears not only to be present but possibly at higher concentration than the mutant allele, and Southern analysis of this same DNA sample supported this observation (data not shown). Thus, in this particular tumor the mutant allele may have been lost and only the wild-type allele retained.

In mice, a tumor cell line has been described in which one copy of an RP gene is deleted and the other copy has suffered a mutation that may contribute to tumorigenesis (Beck-Engeser et al. 2001). To rule out the acquisition of a point mutation in the wild-type allele in RP mutant tumors, all of the coding exons of the appropriate RP gene and at least 50 bp of intronic sequence flanking them were sequenced from each normal and tumor DNA sample. There was no indication of any point mutations in any of the tumors. The apparent retention of the wild-type allele in the tumor cells in these samples, and the fact that no point mutations were observed in the wild-type RP genes in the tumor cell DNA, suggests that it is not a second hit in these loci that leads to tumorigenesis. Rather, the data obtained suggest that these genes function as haploinsufficient tumor suppressors in zebrafish.

### RP Mutations Alter the Relative Amounts of 18S and 28S rRNAs

**Figure 5:**
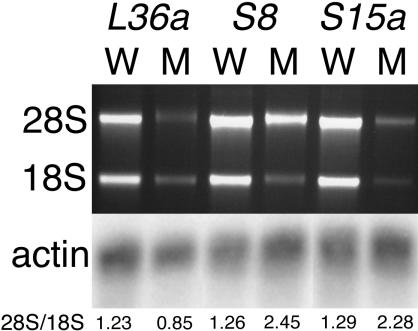
Ribosomal RNA Levels Are Reduced in RP Mutants RNA was prepared from 3-d-old homozygous mutant embryos or their wild-type siblings from lines hi10 *(L36a),* hi1974 *(S8),* and hi2649 *(S15a),* and RNA content was visualized by electrophoresis and ethidium bromide staining. The ratio of 28S/18S as determined by densitometry is shown below each lane. Note that *L36a* mutants show a preferential loss of the 28S band by 1.5-fold, while *S8* and *S15a* mutants show a preferential loss of the 18S band by 1.9- and 1.8-fold, respectively. These RNAs were also northern blotted and probed for beta actin as an mRNA content control.

In yeast, a decrease in the amount of at least some RP genes results in a reduction in the amount of the corresponding ribosomal subunit and a reduction in the number of assembled ribosomes (Moritz et al. 1990). To determine if this is also true in fish, we examined the relative amounts of 18S and 28S rRNA in homozygous mutant embryos compared to sibling controls. Embryos from heterozygote crosses of lines hi10, hi1974, and hi2649 were sorted by phenotype at 3 d post-fertilization, and total RNA was prepared from pools of mutant or phenotypically wild-type sibling embryos. Electrophoresis and ethidium bromide staining were used to determine the amounts of 18S and 28S RNA, which we assume reflect the amounts of 40S and 60S ribosomal subunits, respectively (Figure 5). As a loading control, the same RNA samples were subjected to Northern analysis and probed for beta actin (Figure 5). In each case we observed a decrease in the overall amount of rRNA, and, significantly, a preferential loss of the rRNA found in the ribosomal subunit with which the mutated RP was associated. Thus in hi10, in which a component of the large ribosomal subunit was mutated, while both 18S and 28S RNA levels were decreased, the level of 28S RNA was affected more than that of 18S. Conversely, in hi1974 and hi2649, in which components of the small ribosomal subunit were mutated, the 28S RNA levels were mildly reduced, but 18S RNA was sharply decreased. In none of these cases was the actin level reduced, so the effect was not simply a result of a reduction of cell number, RNA degradation, or cell death. Thus, as in yeast, RP mutations in fish that result in reduced gene expression lead to a relative decrease in the amount of the subunit to which they belong as measured by a decrease in rRNA.

## Discussion

In this study, we have found that heterozygous mutations in 11 different ribosomal protein genes predispose zebrafish to cancer, predominantly to zMPNSTs, but also to other rare tumor types. All of these mutations reduce RP gene expression, indicating that these 11 genes are not oncogenes. Moreover, in the tumors we examined, the wild-type allele appeared to be present and did not contain point mutations; thus these genes are not recessive tumor suppressors. Rather, our findings suggest that these 11 genes are haploinsufficient tumor suppressor genes; that is, reducing their activities by about a factor of two increases the likelihood of cancer. These findings raise two important, unanswered questions: first, how do these mutations lead to cancer, and second, do similar mutations cause cancer in humans?

### How Do These Mutations Cause Cancer?

The finding that mutations in so many different RP genes, including *S7, S8, S15a, S18, S29, L7, L13, L23a, L35, L36,* and *L36a,* predispose to cancer suggests that a function shared by RPs underlies their role in this phenotype. However, not all RP genes were cancer genes: *S12, S15, L3, L24,* and *LP1* heterozygotes appeared normal. This raises the possibility that the oncogenic RP genes could conceivably share some novel biological function independent of their role in the ribosome and that inhibition of this function leads to tumor formation. Individual RPs have been implicated in a wide variety of biological functions, including cell cycle progression, apoptosis, and DNA damage responses (Ben-Ishai et al. 1990; Sonenberg 1993; Chen et al. 1998; Chen and Ioannou 1999; Hershey and Miyamoto 2000; Volarevic et al. 2000; Volarevic and Thomas 2001; Lohrum et al. 2003), and it has been suggested that their role in these processes may arise independently of their role in the ribosome itself (Wool 1996; Wool et al. 1996; Soulet et al. 2001). However, it seems somewhat unlikely to us that there could be such an important, yet still undetected function involving so many different RPs. Thus, we favor the possibility that it is a shared, ribosome-associated function that allows them to be tumor suppressors. If so, then why were not all RP genes cancer genes in this study? At present we can only speculate. We have not found any correlation that distinguishes the RP genes that predispose to cancer from those that do not. Both can belong to either the large or the small ribosomal subunit, and all the mutants show reduced gene expression. Possibly some RP genes are normally expressed at higher levels than others, so that a 50% reduction in their expression does not reduce their protein level below some critical, hypothetical threshold required for tumor suppression.

The best-known function shared by RPs is their role in the assembly of ribosomal subunits, and as a result, their role in translation. In homozygous mutant fish embryos, the RP mutations reduce the amount of the rRNA of the subunit to which they belong, and hence almost certainly reduce the amount of the corresponding ribosomal subunit relative to the remaining subunit. In yeast this is known to reduce the number of ribosomes, and thus also to reduce the amount of protein synthesis. How might this predispose to cancer? In truth, we do not know, and suspect that understanding the mechanism that explains these findings will lead to new insights into growth control. At present we can only list our speculations and several relevant observations.

Reduced protein synthesis could lead to a reduction in the level of a critical tumor suppressor protein, or of a positive regulator of apoptosis or differentiation, either of which could favor growth. A reduction in ribosome number might signal the cell to try to overcome the deficit by making more of the components required for ribosome biogenesis, and this in turn might promote cell growth. Alternatively, a reduction in the number of ribosomes might alter the identity of the messages recruited to ribosomes—similar to the way that modulation of the translational capacity of mammalian cells by oncogenes such as *Ras* or *Akt* is known to alter the identity of mRNAs recruited to polysomes—changing the translation rate of growth-promoting genes (Rajasekhar et al. 2003). Finally, and most speculative of these possibilities, reduced ability of a ribosomal subunit to assemble properly might generate a signal that cells interpret as growth-promoting. For example, degradation of excess rRNA, a molecule with many hairpins, might generate such a signal in the form of RNAi.

### Are RP Genes Cancer Genes in Other Vertebrates?

Given that so many different RP genes can be cancer genes in fish, it seems surprising that they are not already a well-known class of cancer genes in vertebrates. Only two examples are known that suggest a role for RP mutations in mammalian tumor susceptibility, one in mice and one in humans. In the mouse study, two independent murine tumor cell lines were found to express tumor antigens that were mutated RPs (Beck-Engeser et al. 2001). In both cases, the tumors were found to become more aggressive upon either loss or mutation of the wild-type allele of the RP gene. It was postulated that the mutant RPs might have an oncogenic activity that was suppressed by the wild-type protein. Such a mechanism does not seem to be involved in the tumors that develop in the RP mutant fish described here, since we failed to detect evidence of oncogenic activation of RP genes.

In humans, there is a possible association of mutations in one particular RP gene with cancer: approximately 25% of both sporadic and familial cases of Diamond-Blackfan anemia (DBA) are associated with a mutation of *rpS19* (Draptchinskaia et al. 1999), and this syndrome includes an increased risk of developing leukemia (Wasser et al. 1978). It has been demonstrated that the anemia is likely due to a block in erythroid differentiation (Hamaguchi et al. 2002), but it is currently unclear whether the leukemia is an indirect result of the anemia, caused by a stimulation in the production of hematopoietic precursors, or whether the *rpS19* gene dosage plays a direct role in tumorigenesis. It is important to note that DBA is a multigenic disease with very heterogeneous clinical presentation. While DBA patients in general have an increased predisposition to certain cancers, it is not yet clear whether this is true of the subset whose DBA is caused by *rpS19* mutation.

While these examples from mouse and human are consistent with the idea that mutations in individual RP genes might contribute to tumorigenesis in mammals, they have seemed to be unusual examples, rather than suggesting that RP genes in general might be potential cancer genes. Our study suggests for the first time, we believe, that this is a general property of many RP genes. The possibility that a reduction in ribosome levels might be oncogenic in mammals is further supported by the fact that mutations in DKC1, a pseudouridine synthase that is required for rRNA processing and for properly functioning ribosomes, cause dyskeratosis congenita, a disease characterized by both premature aging and increased tumor susceptibility (Ruggero et al 2003).

If RP genes frequently cause human cancers, it is not at all certain that their role would have been detected. Even a deliberate search for their involvement in human cancers would be difficult because there are so many (80) RP genes. This plethora of genes, the fact that it is hard to know which tumor type(s) to examine for RP mutations, and the fact that the mutations might lie in regulatory elements rather than protein-coding regions of the genes would make such a search difficult. Nonetheless, given the high degree of conservation of biological mechanisms among vertebrates, it seems likely that *rp* mutations will prove to increase the incidence of tumors in humans as they do in zebrafish. If so, it may be advantageous to devise diagnostic strategies based on ribosomal protein levels or on a function that these proteins share, for example, in translation, rather than on the analysis of such a large number of individual genes.

In summary, by examining aging populations of mutant lines of fish with defects in embryonic essential genes, we identified a novel group of cancer genes. The ability to identify cancer genes by screening populations of fish heterozyogous for recessive embryonic mutations and the reassuring finding that *NF2a* is a tumor suppressor gene in this system demonstrate the power of large-scale, forward-genetic screens in the zebrafish to identify new disease susceptibility genes.

## Materials and Methods

### 

#### Mutagenesis and maintenance of mutant lines

The insertional mutagenesis screen was carried out as previously described (Amsterdam et al. 1999). Stocks of all lines were maintained by outcrossing heterozygotes to nontransgenic fish, preparing DNA from tail fin biopsies of 8- to 18-wk-old fish, and performing PCR with insert-specific primers for each line to identify heterozygotes.

#### Fixation and histology

Adult fish were euthanized in ice water and fixed within 30 min in Bouin's solution, embedded in paraffin, and sectioned as previously described (Moore et al. 2002).

#### LOH analysis

DNA was prepared from tumor tissue or tail tissue isolated from fish prior to fixation for histology. PCR was conducted with one primer complementary to proviral sequence and two primers complementary to sequences on either side of the insertion for the appropriate mutation. Primer sequences were as follows: hi10: 10gen5 (5′-CAGCACAGATTCTTGAAAGCGCC-3′), 10gen3 (5′- GCATATGTAGCATCTCGAAGGTCC-3′), and NU3X (5′- TGATCTCGAGCCAAACCTACAGGTGGGGTC-3′); hi258: 258A5a (5′-GGTACGTCTGTGCTTATGTTTGTGTC-3′), 258A3a (5′-TCTCAAGACTTCATCCATTCATAATTCTGC-3′), and NU3X; hi1974: 1974c1 (5′-CTACACCACAGGTATCTCAAGGG-3′), 1974c1est3 (5′-CCACCACGGACTCTTATTGTGTG-3′), and IPL3 (5′-TGATCTCGAGTTCCTTGGGAGGGTCTCCTC-3′).

#### RNA analysis

RNA was prepared from mutant and wild-type embryos using Trizol reagent (Invitrogen, Carlsbad, California, United States). For RT-PCR, serial dilutions of first strand cDNA were amplified for 30 (hi1974) or 35 (hi10 and hi258) cycles using the following primers for the genes indicated: *rpL36a:* 10rt5 (5′-CAACCATGGTAAACGTACCGAAG-3′) and 10RTR (5′-CACAAAAGAAGCACTTGGCCCAGC-3′); *rpL35:* 258RTF2 (5′-GCTGCTTCCAAGCTCTCAAAAATCC-3′) and 258RTR (5′-TGCCTTGACGGCGAACTTGCGAATG-3′); *rpS8:* 1974RTF1 (5′-TCTCAAGGGATAACTGGCACA-3′) and 1974RTR1 (5′-GAACTCCAGTTCTTTGCCCTC-3′); actin: actinF (5′-CATCAGCATGGCTTCTGCTCTGTATGG-3′) and actinR (5′-GACTTGTCAGTGTACAGAGACACCCT-3′). For visualization of 18S and 28S RNA, two embryo equivalents of RNA were electrophoresed through a nondenaturing agarose gel containing 0.5 μg/ml ethidium bromide. For detection of beta actin RNA, four embryo equivalents of RNA were electrophoresed through a 7.5% formaldehyde/MOPS-buffered agarose gel, blotted to Hybond N+ (Amersham Biosciences, Little Chalfont, United Kingdom), and hybridized with a random primed beta actin probe.

## Supporting Information

Figure S1 Position of Mutagenic Insertions The genomic sequence of part of each of these genes is represented as exonic (boxed) and promoter or intronic (line). White boxes represent 5′ UTR while shaded boxes represent coding exons. Where no white boxes are shown, the location of the 5′ UTR and beginning of the coding region has not been determined relative to the part of the locus shown here. In all cases, at least one coding exon (and all of the 3′ UTR) is downstream of the region of the gene represented here. The position and orientation of the proviruses are shown above each genomic sequence. All drawings are to the scale of the top scale bar, except the *rpl36* locus, which has its own scale bar.
(62 KB PDF).

### Accession Numbers

The GenBank (http://www.ncbi.nlm.nih.gov/Genbank/) accession numbers for the genes discussed in this paper are *L13* (AY561516), *L23a* (AY561517), *L24* (AY099532), *L3* (AY561514), *L35* (AF506205), *L36* (AY561518), *L36a* (AY099511), *L7* (AY561515), *LP1* (AY561519), *NF2a* (AY561520), *S12* (AY561510), *S15* (AY561511), *S15a* (AY561512), *S18* (AY099517), *S29* (AY561513), *S7* (AY561508), and *S8* (AY561509)*.*

## Abbreviations

DBA: Diamond-Blackfan anemia
LOH: loss of heterozygosity
MPNST: malignant peripheral nerve sheath tumor
NF2: neurofibromatosis type 2
RP: ribosomal protein
zMPNST: zebrafish malignant peripheral nerve sheath tumor
