# Supplementary figures and images for: Many Ribosomal Protein Genes Are Cancer Genes in Zebrafish
Source: PLoS Biol. 2004 May 11;2(5):e139. doi: 10.1371/journal.pbio.0020139 (PMC406397; doi:10.1371/journal.pbio.0020139)

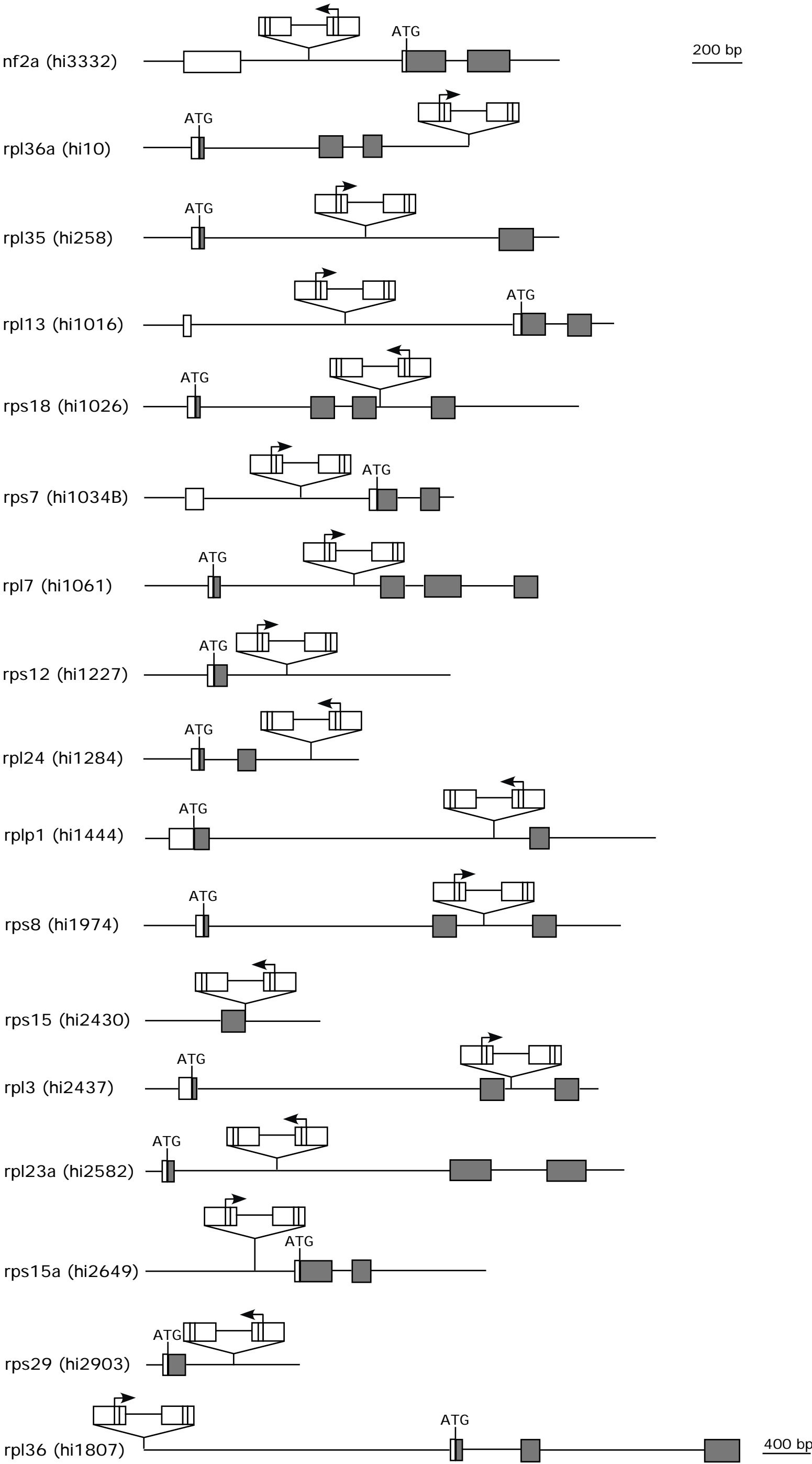

Supplement: Figure S1 — The genomic sequence of part of each of these genes is represented as exonic (boxed) and promoter or intronic (line). White boxes represent 5′ UTR while shaded boxes represent coding exons. Where no white boxes are shown, the location of the 5′ UTR and beginning of the coding region has not been determined relative to the part of the locus shown here. In all cases, at least one coding exon (and all of the 3′ UTR) is downstream of the region of the gene represented here. The position and orientation of the proviruses are shown above each genomic sequence. All drawings are to the scale of the top scale bar, except the rpl36 locus, which has its own scale bar. (62 KB PDF). [file pbio.0020139.sg001.pdf]
